# Development of *Wolffia arrhiza* as a Producer for Recombinant Human Granulocyte Colony-Stimulating Factor

**DOI:** 10.3389/fchem.2018.00304

**Published:** 2018-07-25

**Authors:** Pavel Khvatkov, Alexsey Firsov, Anastasiya Shvedova, Lyubov Shaloiko, Oleg Kozlov, Mariya Chernobrovkina, Alexander Pushin, Irina Tarasenko, Inna Chaban, Sergey Dolgov

**Affiliations:** ^1^Laboratory of Plant Gene Engineering, All-Russia Research Institute of Agricultural Biotechnology, Russian Academy of Sciences, Moscow, Russia; ^2^Sector of Plant Bioengineering, Nikita Botanical Gardens – National Scientific Centre, Russian Academy of Sciences, Yalta, Russia; ^3^Laboratory of Expression Systems and Modification of the Plant Genome “BIOTRON”, Branch of Shemyakin and Ovchinnikov Institute of Bioorganic Chemistry, Russian Academy of Sciences, Puschino, Russia

**Keywords:** biopharming, hG-CSF, recombinant proteins, transgenic duckweed, transgene expression system, *Wolffia arrhiza*

## Abstract

To date, the expression of recombinant proteins in transgenic plants is becoming a powerful alternative to classical expression methods. Special efforts are directed to the development of contained cultivation systems based on cell culture or rhyzosecretion, which reliably prevents the heterologous DNA releasing into the environment. A promising object for the development of such systems is the tiny aquatic plant of *Wolffia arrhiza*, which can be used as a dipped culture in bioreactors. Herein we have expressed the human granulocyte colony-stimulating factor (hG-CSF) in nuclear-transformed *Wolffia*. The nucleotide sequence of hG-CSF was optimized for expression in *Wolffia* and cloned into the vector pCamGCSF downstream of double CaMV 35S promoter. *Wolffia* plants were successfully transformed and 34 independent transgenic lines with hG-CSF gene were obtained, PCR and Southern blot analysis confirmed the transgenic origin of these lines. Western blot analysis revealed accumulation of the target protein in 33 transgenic lines. Quantitative ELISA of protein extracts from these lines showed hG-CSF accumulation up to 35.5 mg/kg of *Wolffia* fresh weight (0.194% of total soluble protein). This relatively high yield holds promise for the development of *Wolffia*-based expression system in strictly controlled format to produce various recombinant proteins.

## Introduction

Nowadays a large number of expression systems for recombinant proteins production have been developed. Recombinant proteins are produced in the cells of bacteria, yeast, mammals, and insects (Walsh, 2014; Rader and Langer, 2015; Sysuev and Pokrovskaya, 2015). However, along with the advantages these systems have a number of substantial drawbacks, in particular post-translation modifications and correct folding of many eukaryotic proteins do not occur in bacterial and yeast cells (Jensen, 2006; Merlin et al., 2014; Tschofen et al., 2016). Animal cells are devoid of such shortcomings but their use as bioproducers is limited by reason of recombinant proteins high production cost (Demain and Vaishnav, 2009). Thus, the development of expression systems combining the advantages of microbial systems (high expression level, relatively low production cost) and systems based on animal cell culture (the complete identity of recombinant protein and its properties to native counterpart) is still urgent.

Apparently the advantages of microbial and animal cell culture expression systems are combined in plant-based platforms to the greatest extent. Basically the protein glycosilation and folding in the higher plants are similar to those in mammalian cells (Strasser, 2016). Unlike animals, plant cells do not contain viruses and microorganisms pathogenic for humans, that substantially simplifies the purification of recombinant proteins for medical purposes (Daniell et al., 2001). Plant cultivation does not require expensive equipment, wherein the agricultural scale of the production guarantees the availability of recombinant proteins in the quantities quite enough for wide therapeutical use (Kaufman, 2011; Wilken and Nikolov, 2012).

The most common current expression systems are based on well-studied agricultural plants, such as tobacco, rice, corn etc. The main problem of these systems is the probability of uncontrolled transfer of heterologous DNA into the environment by seeds, pollen or plant residues during the cultivation of transgenic plant in the field or greenhouse. The fear of this greatly complicates the commercialization of plant-based expression systems. Considerable effort, therefore, has been focused on the development of contained cultivation systems, such as axenic culture of suspension cells or hairy roots (Georgiev et al., 2012; Santos et al., 2016). The contained plant systems can be cultivated in bioreactors, entirely preventing the accidental release of genetically modified plant material to the environment.

Another approach to the development of contained cultivation systems is the using of small aquatic plants—duckweed [*Lemna minor* (L.) and *Wolffia arrhiza* (L.) Horkel ex Wimm.] as recombinant proteins producers. Duckweed are small monocotyledonous plants with a high content of total protein (up to 45% of DW), demonstrating high growth rate (doubling of biomass in 1–6 days) and almost exclusively vegetative reproduction (Armstrong and Thorne, 1984). These plants are capable to photosynthesis *in vitro* and can be effectively and inexpensively cultivated in bioreactors of various types (Gasdaska et al., 2003; Khvatkov et al., 2013).

In *Lemnaceae* family, *Wolffia arrhiza* is the most evolutionary advanced secondary primitively organized species (Wolff, 1992). Plant bodies are tiny; generally globoid to ovoid-ellipsoid or cylindrical; 0.4–1.3 mm long and 0.2–1.0 mm wide; floating on or partially below water surface (Wayne and Thorne, 1984). The most important *Wolffia*'s feature is, that unlike other *Lemnoideae*, it does not have a root system, this property allows the cultivating of *Wolffia* in bioreactors using submergence, which in turn could considerably increase the profitability of recombinant proteins production.

To date, there are already examples of successful expression of commercially important proteins in duckweed plants: the industrial enzyme endoglucanase E1 from *Acidothermus cellulolyticus* (Sun et al., 2007), anti-CD20, anti-CD30, and anti-interferon a2b monoclonal antibodies (Cox et al., 2006; Biolex Therapeutics website), and the protective antigens of influenza virus - peptide M2e (Firsov et al., 2015, 2018), and hemagglutinin (Bertran et al., 2015). Despite that *Wolffia* is very promising plant for biotechnology, yet there is no reports of its use for recombinant proteins expression. The main problem of using *Wolffia* for recombinant proteins expression is the absence of a method for its genetic transformation. The development of such a method allows to start the studies on the development of *Wolffia*—based expression system.

The aim of this study is to explore the feasibility of recombinant proteins expression in nuclear-transformed *Wolffia arrhiza* plants by the example of human granulocyte colony-stimulating factor (hG-CSF). hG-CSF consists of 207 amino acid residues, including a signal peptide (aa 1–29). Mature hG-CSF has a length of 178 aa, it is stabilized by 2 disulfide bonds and glycosylated at position 166. At the present time recombinant hGCSF is widely used for management of various etiology neutropenias in the therapy of patients with HIV, aplastic anemia, pneumonia, leukemia, and others diseases (Harousseau et al., 2000; Babalola et al., 2004; Dale, 2010).

Here, we report the successful Agrobacterial transformation of *Wolffia* and expression of human granulocyte colony-stimulating factor in transgenic plants. Recombinant GCSF accumulated at a high level, sufficient for further studies to create *Wolffia*-based expression system to produce the offered and other recombinant proteins.

## Materials and methods

### Gene synthesis and plasmid construction

The amino acid sequence of hG-CSF corresponding to the drug Filgrastim (DrugBank: DB00099) was selected for expression in *Wolffia*. The N-terminal signal peptide hG-CSF has been replaced by a corresponding peptide of rice α-amilase (GenBank: AAA33897.1). To optimize the codon composition a table of codon usage in *Lemna gibba* was used (http://www.kazusa.or.jp/codon). The optimization of *hG-CSF* nucleotide sequence was performed by Gene Composer software (Lorimer et al., 2009). The set of overlapping oligonucleotides was designed using Gene2Oligo (Rouillard et al., 2004).

Nucleotide sequence of hG-CSF was synthesized by PCR (Rouillard et al., 2004) and cloned into sites HindIII and EcoRI of plasmid pUC18 followed by sequencing. Then, hG-CSF (in Appendix) sequence was cut out from pUC18 and, using the same sites, cloned into plant expression vector pCamPPVcp, replacing the plum pox virus coat protein gene (Dolgov et al., 2010). The resulting plasmid pCamGCSF was transferred into *A. tumefaciens* strain EHA105 (Hood et al., 1993) and used for *Wolffia* transformation.

### Tissue culture and *Agrobacterium*-mediated transformation of *W. arrhiza*

**Figure 1:**
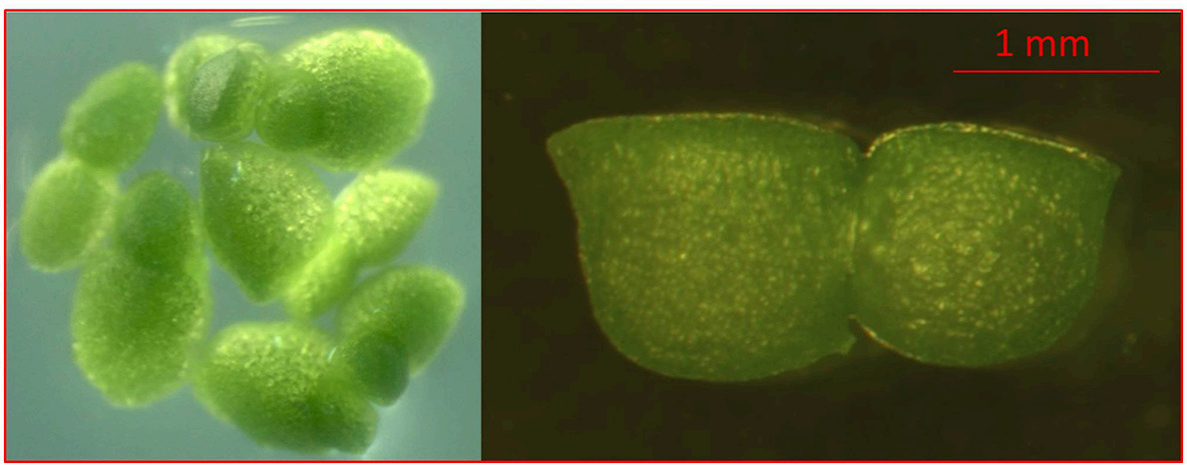
Images of characteristic morphological features of *Wolffia arrhiza*. Dorsal view showing several flat-topped, dark green plants and side view of *W. arrhiza* budding plant showing flattened, dorsal surface of daughter plant.

For research, we obtained a population of *Wolffia arrhiza* (L.) Horkel ex Wimm from the collection of Main Botanic Garden of the Russian Academy of Sciences (RDSC Clone *Wolffia* 5564; Supplementary Table 1) (Figure 1).

An aseptic population of *W. arrhiza* plants was cultivated on SH medium (Schenk and Hildebrandt, 1972) supplemented with 2% (w/v) sucrose and 0.7% (w/v) agar (Panreac, Spain). 10 plants were placed on each Petri dish filled with culture medium. The cluster structures formed after 4 months of pre-cultivation on MS2G media containing 5.0 mg l^−1^ 2,4-Dichlorophenoxyacetic acid (2,4-D) and 0.5 mg l^−1^ N^6^-Benzyladenine (BA) (Khvatkov et al., 2015b), were used for transformation. The explants developed at light intensity of 65 μmol m^−2^ s^−1^, day photoperiod 16 h and temperature 21 ± 1°C.

*W. arrhiza* was transformed as described by Khvatkov et al. (2015a). Explants were co-cultivated with *A. tumefaciens* containing the plasmid pCamGCSF, with subsequent transfer to SH medium containing 2.0 mg l^−1^ 2,4-D, 2.0 mg l^−1^ BA, 5.0 mg l^−1^ hygromycin B (Hyg) and 100 mg l^−1^ timentin. After2 weeks the explants were transferred to SH medium lack of growth regulators for regeneration. Each newly emerged Hyg-resistant plant was placed into a separate culture tube with W3M media (Dolgov et al., 2013) supplemented with 5.0 mg l^−1^ Hyg for further development.

The inspection of the selected plant material for their regenerative capacity [viz. the development of meristematic tissue (Figure 3a) and formation of plant regenerants (Figure 3b)] was performed by light microscopy. Preparation of samples and microscopic examination of plant material was carried out according to the procedure described in Miroshnichenko et al. (2017).

Selection of pure transgenic lines was carried out as described by Khvatkov et al. (2015a). Transformation efficiency was determined by dividing the number of explants which formed transgenic populations upon the total number of explants in each experiment (percentagewise).

### PCR and southern blot analysis of transformants

The *Wolffia* genomic DNA was isolated from transformed and non-transformed plants according to the method of Dellaporta et al. (1983). The respective forward and reverse primer sequences for *hpt*II were hptf: 5′-acattgttggagccgaaatc-3′ and hptr: 5′-gacattggggagtttagcga-3′; those for *hG-CSF* were G1for: 5′-gtccaagcttatggcgaagaggatcgcc-3′ and G2rev: 5′-atgaattctcacggttgggcgagatg-3′; those for *virC* were virC1: 5′-gcactatctacctaccgctacgtcatc-3′ and virC2: 5′-gttgtcgatcgggactgtaaatgtg-3′.

For Southern blot analysis, genomic DNA from transformed and non-transfomed *Wolffia* plants in amount of 50 μg was digested overnight at 37°C with 100 U HindIII which cuts the T-DNA of pCamGCSF at a single position. After agarose gel electrophoresis (0.8%), the products of digestion were transferred and immobilized onto Hybond+ membrane (Amersham, USA) according to the manufacturer's instructions. The DNA probe was constructed by PCR using plasmid pCamGCSF as the template, and primers G1for and G1rev. Probe DNA (600 bp) was labeled with alkaline phosphatase using the AlkPhos Direct Labeling Kit (Amersham Bioscience, USA). Prehybridization, hybridization (overnight at 60°C) with alkaline phosphatase-labeled probe, and subsequent washings of the membrane were carried out according to the AlkPhos Direct Labeling Kit protocol. Detection was performed using CDP-Star detection reagent according to the manufacturer's instructions (Amersham Bioscience).

#### Western blot analysis

Transgenic and non-transformed *Wolffia* plants (in an amount of 1 g) were grinded in liquid nitrogen and resuspended in 3x volume extraction buffer (50 mM Tris-HCl (pH7.6), 150 mM NaCl, 5 mM EDTA, 5 mM β-merkaptoethanol, 0.62 μM aprotinin, 8.4 μM leupeptin). Extraction was performed during 40 min at 4°C followed by centrifugation for 20 min at 16,000 g; supernatant was collected for subsequent analysis. Protein concentration in the samples was determined by Bradford method (Bradford, 1976).

Protein electrophoresis (70 μg/lane) was carried out in 10–25% gradient SDS-PAGE. The separated proteins were transfered onto a nitrocellulose membrane (BioRad, USA). Rabbit polyclonal antibody against hG-CSF (diluted 1:1,000; Abcam, UK) and anti-rabbit IgG conjugated with alkaline phosphatase (1:3,000; Pierce, USA) were used. Recombinant hG-CSF (Abcam, UK) served as the positive control. The blots were visualized using chromogenic substrate BCIP/NBT (Fermentas, Lithuania).

#### ELISA quantification of hG-CSF accumulation

The total protein was extracted as described above. The protein samples were serially diluted in PBS and loaded into 96-well ELISA plate (0.25, 0.5, 1.0, and 2.0 μg of TSP per well), using recombinant hG-CSF (Abcam, UK) as the reference standard.

The plates were incubated for 2 h at RT, then washed three times with PBST (PBS with 0.1% Tween 20) for 5 min each and blocked in PBST containing 2% bovine serum albumin (1 h at RT). Hybridization with the primary antibody was performed overnight at 4°C. After washing, the secondary antibody was added and plates were incubated for 1 h at RT followed by the washing. Rabbit polyclonal antibody against hG-CSF (Abcam, UK) was diluted at 1:1,000, anti-rabbit IgG conjugated with alkaline phosphatase (Pierce, USA)—at 1:2,000. The plates were developed for 30 min at RT using TMB Peroxidase EIA substrate (BioRad). The plates were read at 405 nm and the amount of plant—expressed hG-CSF was estimated based on reference standards.

#### Statistical analysis

The significance of differences in hG-CSF accumulation between *Wolffia* transgenic lines were analyzed by analysis of variance (ANOVA) followed by multiple comparisons of individual averages and evaluation by Duncan's test (Statistica 6.1 software; StatSoft Inc).

## Results

### Vector construction and *Agrobacterium*-mediated transformation of *Wolffia arrhiza*

**Figure 2:**
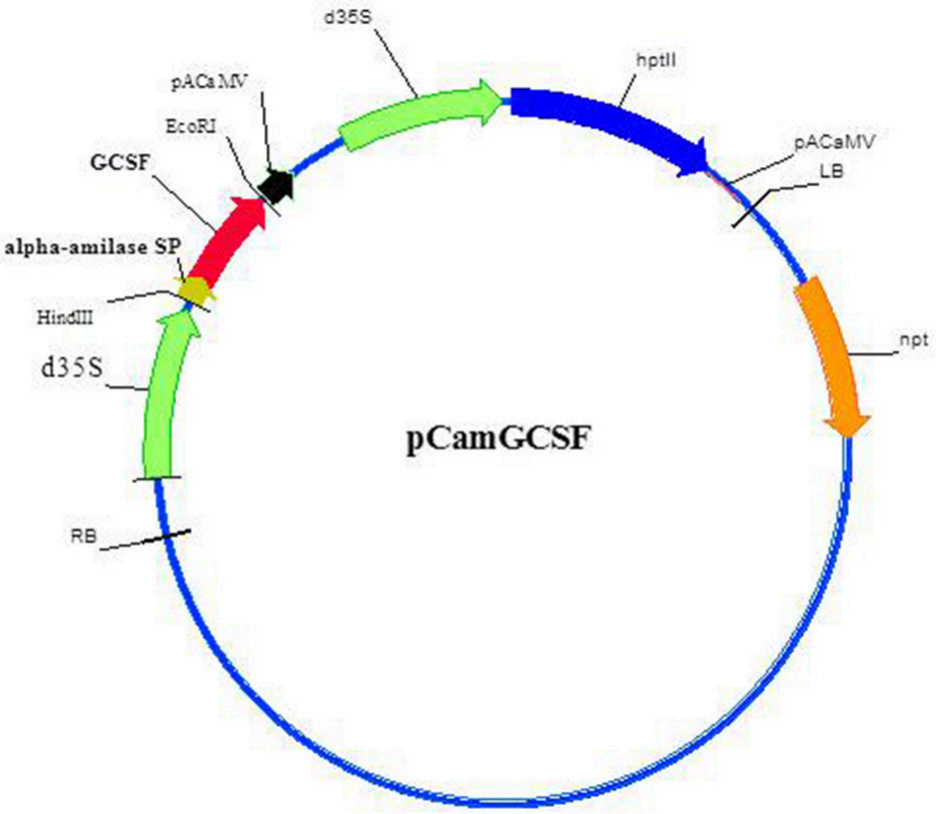
Schematic presentation of pCamGCSF plasmids used for *Wolffia* transformation. d35S, double 35S RNA Cauliflower Mosaic Virus promoter; *GCSF*, human granulocyte colony-stimulating factor coding sequence; *alpha-amilase SP*, nucleotide sequence of rice alpha-amylase signal peptide; *hpt*ll, hygromycin phosphotransferase (HPT) coding sequence; pACaMV, 35S RNA Cauliflower Mosaic Virus terminator with polyadenylation signal; RB, right border; LB, left border; *npt*, neomycin phosphotransferase (NPT) coding sequence allowing for *A. tumefaciens* selection.

We used the data on codon usage in *Lemna gibba*, which belongs to the same *Lemnaceae* family, as *W. arrhiza*, to optimize the codon composition of *hG-CSF* nucleic acid sequence. We assumed that the codon usage do not differ significantly in these two species. Nucleic sequence of *hG-CSF* with the codon composition optimized for the expression in *Lemnaceae* was synthesized and cloned under the control of the CaMV double 35S promoter. The resulting plasmid pCamGCSF (Figure 2) was used for hG-CSF expression in *Wolffia* plants, following its *Agrobacterium*-mediated transformation.

**Figure 3:**
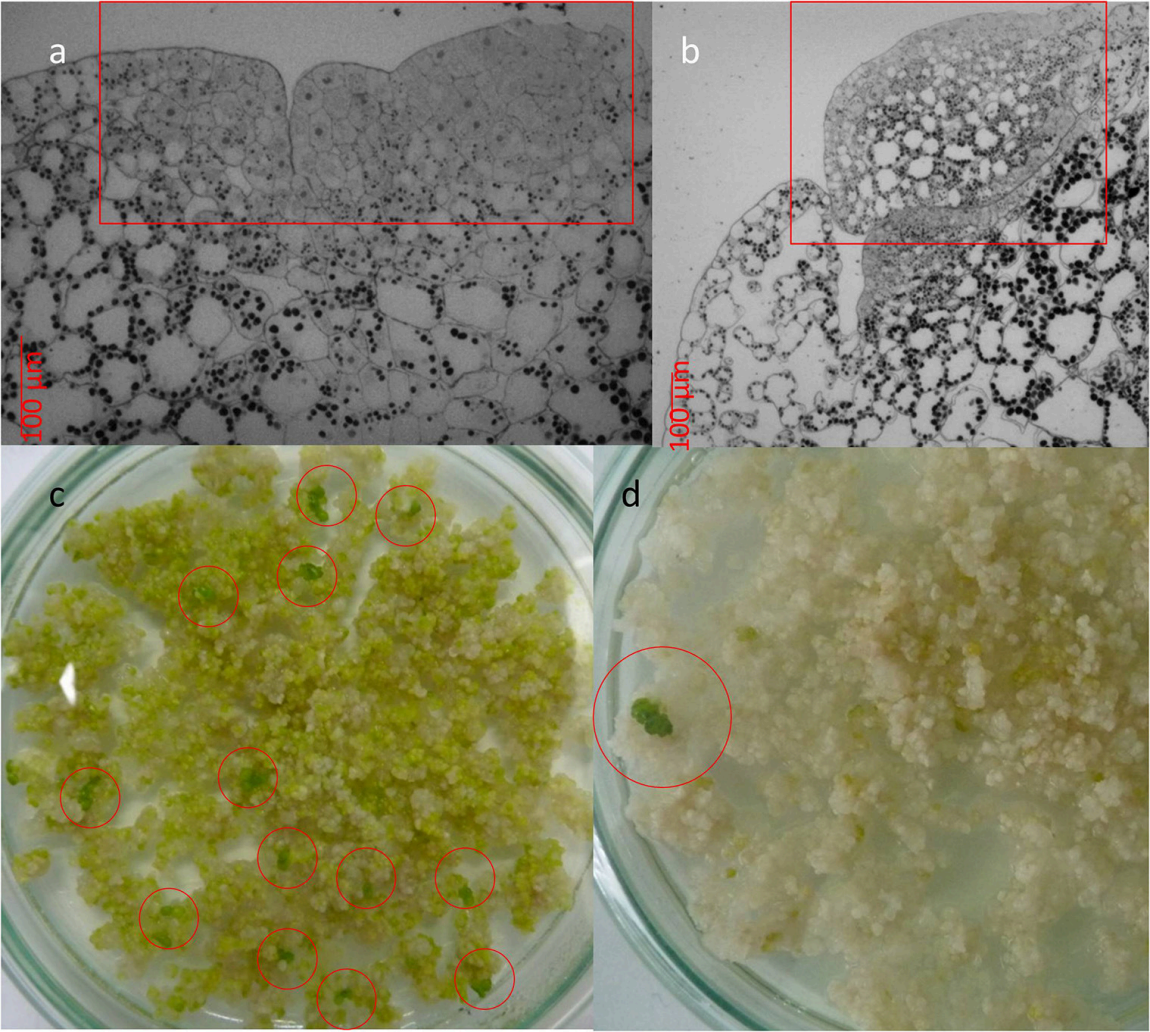
Development of transgenic *Wolffia* plants in the presence of 5.0 mg l^−1^ Hyg in the culture media. **(a)** Meristematic area (squared in red) on the cluster surface which developed after 6 weeks exposure to selective media. **(b)** Globular structures (squared in red) on the cluster surface which developed after 8–10 weeks exposure to selective media. **(c, d)** Multiple **(c)** or single **(d)** formation of whole plants (circled in red) in the presence of 5.0 mg l^−1^ Hyg in the culture media.

In experiments on *Agrobacterium-*mediated transformation, transgenic tissue is distinguishable after 1.5 months under selection on SH medium in the presence of 5.0 mg l^−1^ Hyg. Green meristematic area appeared on cluster surface after 6 weeks of cultivation on SH medium lack of growth regulators (Figure 3a, squared in red). Emerald green globular structures were originally developed from meristematic area (Figure 3a, squared in red) followed by the arising of the whole plants (Figures 3c,d, circled in red). Regeneration of various cultures on the hormone-free medium is a rare but fairly well-known phenomenon. Thus, in addition to the *Wolffia*, the ability to regenerate on hormone-free media is capable, for example, of carrots (Górecka et al., 2009), lotus (Rybczynski and Badzian, 1987), flax (Aycan et al., 2014), parsley (Masuda et al., 2006), beets (Doley and Saunders, 1989), and etc. Obviously, hormone enriched media during the cultivation of explants of these cultures *in vitro* are required solely to maintain them in the state of the callus structures, and not for regeneration.

The morphological differences between Hyg-resistant *Wolffia* plants and non-transformed ones were not observed. In liquid culture the development and growth rate of transgenic plants did not differ from the corresponding characteristics of the non-transformed control plants. In experiments on *Agrobacterium*-mediated transformation of *Wolffia*, 8100 explants were used on the whole; the transformation frequency was 0.42%. In total, 34 independent *Wolffia* lines which developed without signs of hygromycin toxic effect were obtained; these lines were used for further analysis.

### PCR and southern blot analysis of transformants

The fragments of the expected size were amplified from the DNA of all analyzed putatively transgenic *Wolffia* lines. As it was shown by PCR using virC1 and virC2 primers, all lines were free of agrobacterial contamination (data not shown). Thus, according to PCR analysis, all of 34 Hyg-resistant *Wolffia* lines contained selective *hpt*II gene and target hG-CSF sequence.

**Figure 4:**
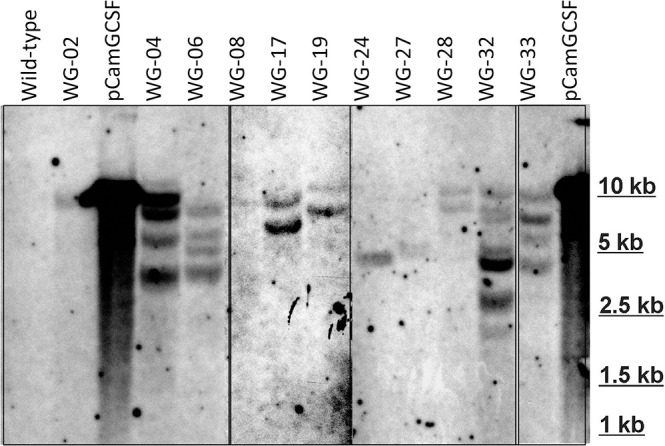
Southern hybridization analysis of some EHA105/pCamGCSF-transformed *Wolffia* plants. DNA (10 μg) digested with HindIII and hybridized with a 600-bp *hG-CSF* probe. pCamGCSF, pDNA (pCamGCSF/HindIII**)**; WG, DNA of transgenic *Wolffia* lines digested with HindIII; Wild-type, genomic DNA of wild-type *Wolffia* plants digested with HindIII.

The selected transgenic lines with different hG-CSF expression were analyzed by Southern blot. The obtained results confirmed integration of hG-CSF gene into *Wolffia* genome (Figure 4). Based on the hybridization profile, there were one (lines 2, 8, 24, and 27), two (lines 17, 19, 28), and four or more insertions (lines 4, 6, 32, and 33) of the transgene into the obtained lines, which is typical for *Agrobacterium*-mediated transformation. The DNA from non-transformed plants failed to hybridize to the probe.

### Analysis of hG-CSF expression in transgenic *Wolffia* plants

**Figure 5:**
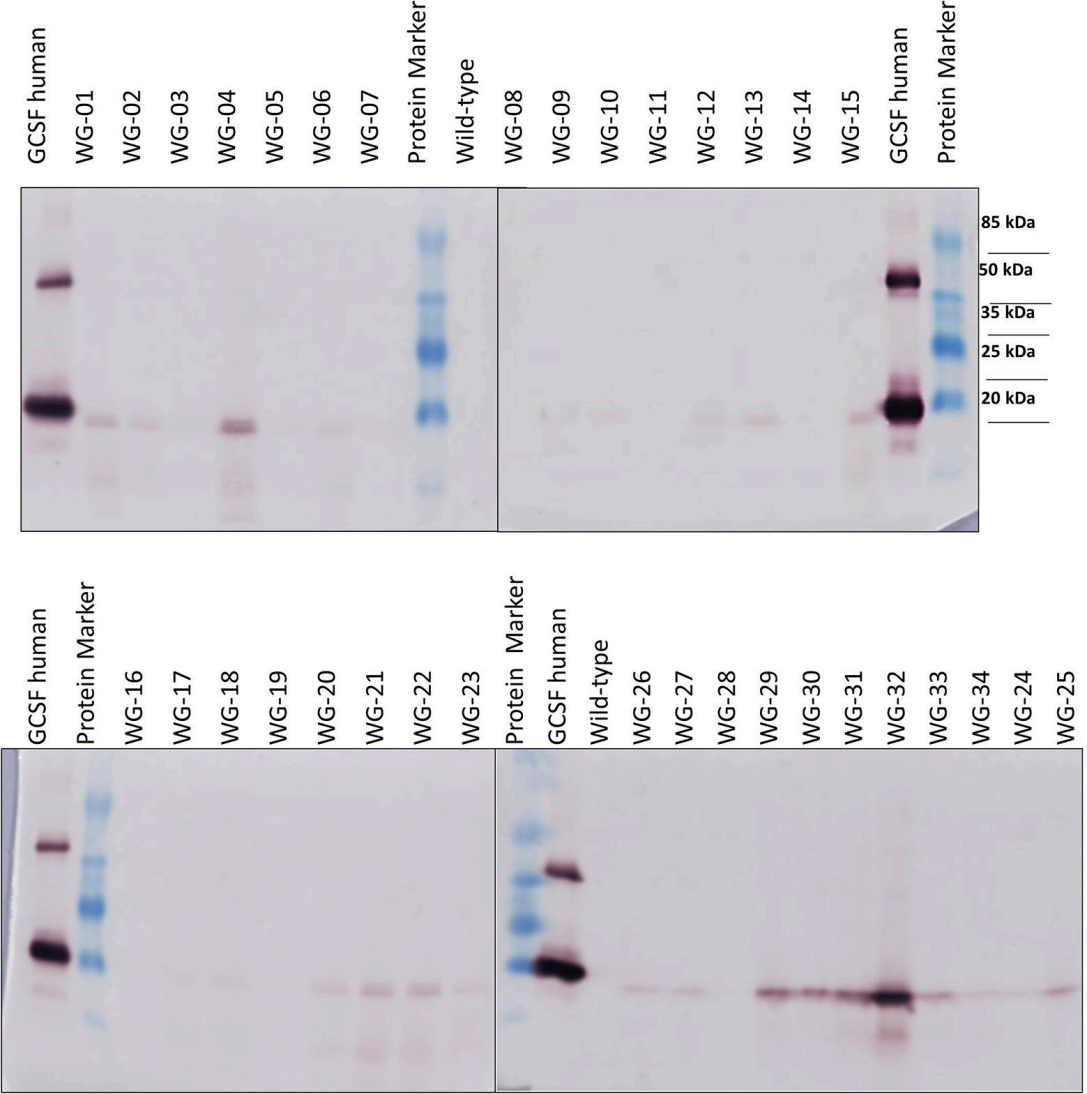
Western blot analysis of hG-CSF expression in some transgenic *Wolffia* lines. Protein Marker, molecular size marker TS 26612 Protein MW Marker; GCSF human, recombinant human G-CSF (200 ng; AbCam, UK).

Western blot analysis using anti-hG-CSF antibody has revealed the presence of a 20-kDa band corresponding to the recombinant hG-CSF in 33 transgenic lines out of 34 examined (Figure 5). hG-CSF from the transgenic plants migrated at the same rate as the control protein. This result confirms the cleavage of the rice α-amylase N- terminal signal peptide, i.e., the correct processing of pre-hG-CSF in *Wolffia* plants. Immunoreactive bands of similar weight were not observed in the protein samples from non-transformed control plants. It should be noted, that there was no noticeable degradation of the recombinant hG-CSF in transgenic plants. In some lines (№ 3, 5, 7, 8, 11, 14, 16, and 19), the target protein was detected at low level; the immunoreactive bands were weak (Figure 5). The maximum hG-CSF accumulation was observed in lines WG-04 and WG-32, in others lines the accumulation of the target protein was lower.

**Figure 6:**
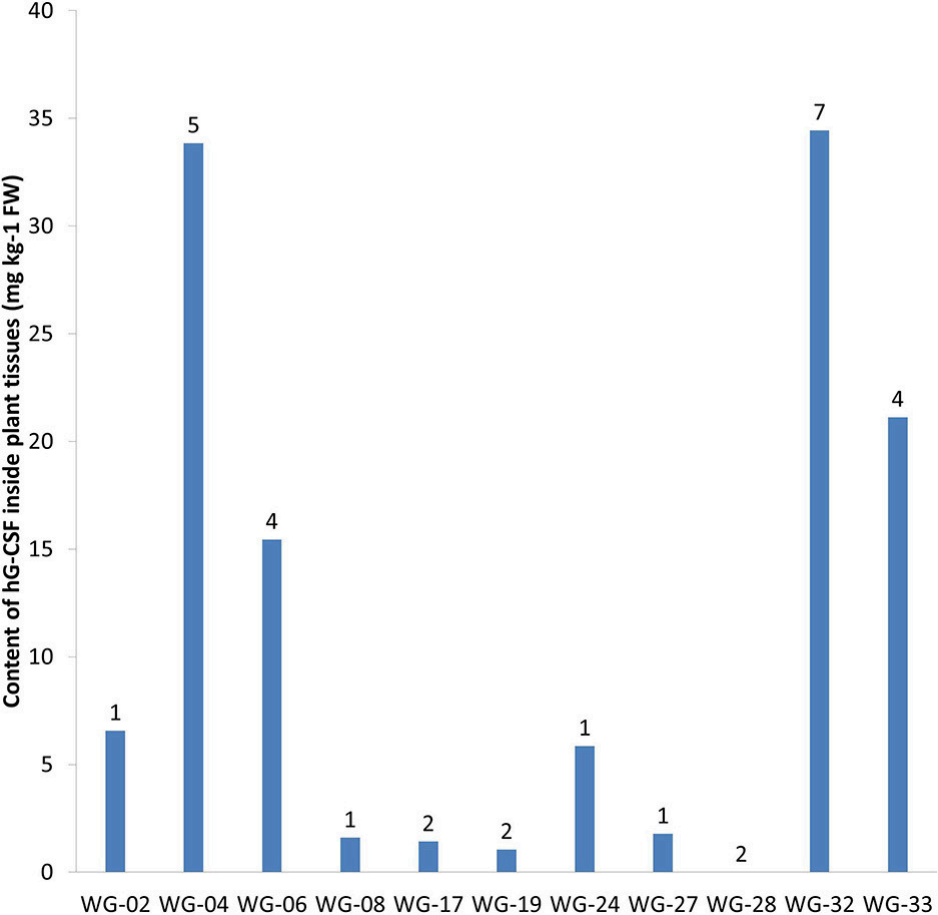
The result of ELISA of 11 transgenic *Wolffia* lines containing *hG-CSF* gene. Numbers designate the number of *hG-CSF* gene insertions into transgenic line genome.

According to ELISA, 33 out of 34 transgenic lines expressed hG-CSF in plant tissues (Table 1). Quantification of the target protein in transgenic *Wolffia* plants using anti-hG-CSF antibody showed accumulation from 0.36 to 35.5 μg of hG-CSF per 1 g fresh weight (FW) of *Wolffia*, corresponding to 0.002–0.194% of total soluble protein (TSP). The highest levels of hG-CSF accumulation were observed in lines WG-04 and WG-32 (33.9 and 35.5 μg g^−1^ FW; 0.191 and 0.194% of TSP, respectively), and the lowest in lines WG-09, WG-13 and WG-14 (0.36, 0.53 and 0.36 μg g^−1^ FW; 0.002, 0.003, and 0.002% of TSP, respectively) (Figure 6).

**Table 1:** Quantitative indicators of hG-CSF expressed in *W.arrhiza* transgenic plants.

**Name of transgenic line**	**Saturation with hG-CSF inside plant tissues**	
	**Target protein per TSP (%)**	**mg kg^−1^ of FW**
WG-01	0.005 ± 0.002	0.95a
WG-02	0.037 ± 0.017	6.57ab
WG-03	0.014 ± 0.006	2.49ab
WG-04	0.191 ± 0.052	33.85de
WG-05	0.011 ± 0.006	1.95a
WG-06	0.087 ± 0.070	15.45bc
WG-07	0.008 ± 0.004	1.42a
WG-08	0.009 ± 0.001	1.60a
WG-09	0.002 ± 0.001	0.36a
WG-10	0.004 ± 0.003	0.71a
WG-11	0.007 ± 0.002	1.24a
WG-12	0.004 ± 0.003	0.71a
WG-13	0.003 ± 0.001	0.53a
WG-14	0.002 ± 0.001	0.36a
WG-15	0.007 ± 0.004	1.24a
WG-16	0.008 ± 0.002	1.42a
WG-17	0.008 ± 0.004	1.42a
WG-18	0.005 ± 0.002	0.89a
WG-19	0.006 ± 0.001	1.06a
WG-20	0.005 ± 0.003	0.89a
WG-21	0.005 ± 0.002	0.89a
WG-22	0.006 ± 0.001	1.07a
WG-23	0.006 ± 0.004	1.0a
WG-24	0.033 ± 0.021	5.86ab
WG-25	0.008 ± 0.001	1.42a
WG-26	0.005 ± 0.003	0.89a
WG-27	0.010 ± 0.001	1.78a
WG-28	0.000 ± 0.000	0.00a
WG-29	0.010 ± 0.004	1.78a
WG-30	0.028 ± 0.008	4.97ab
WG-31	0.006 ± 0.004	1.07a
WG-32	0.194 ± 0.060	35.45e
WG-33	0.119 ± 0.023	15.13c
WG-34	0.004 ± 0.003	0.71a

*Different letters in a column indicate significant differences in variant data according by Duncan's test*.

As a result of our study, our research team was the first to receive transgenic *Wolffia* plants expressing the recombinant human G-CSF. In two lines—WG-04 and WG-32—the target protein was accumulated at a high level—approximately 35 mg of recombinant protein per 1 kg of *Wolffia* FW. Thus, by the example of hG-CSF we have demonstrated the potential of *Wolffia* as a producer of recombinant proteins.

## Discussion

We have obtained 34 different lines of *Wolffia* with confirmed transgenic status; the target protein hG-CSF was detected in 33 lines (Table 1). Morphologically transgenic *Wolffia* plants did not differ from their non-transformed counterparts. Expression of the foreign protein did not affect the rate of reproduction in liquid culture or plant TSP content. According to ELISA accumulation of recombinant hG-CSF ranged from 0.36 to 35.5 μg g^−1^ FW of *Wolffia* (0.002–0.2% of TSP).

In our study, we identified almost 100-fold variation in target protein accumulation among the transgenic lines. Variations in recombinant protein expression between independently derived transgenic lines are common (Richter et al., 2000; Sharma and Sharma, 2009). They are often related to differences in the number of transgene copies or in the position within the genome into which the foreign DNA was integrated (Dolgova et al., 2015). Both of these factors might be applied to the transgenic *W. arrhiza* lines that were obtained (Figure 6). The Southern blot analysis showed notable differences between the studied lines both as in the number of transgene inserts and in the sites of their integration into the plant genome.

Currently the recombinant hG-CSF used in medicine is produced either in *E. coli* expression system (Filgrastim medication; Vacchelli et al., 2013) or in CHO cell culture (Lenograstim; Serova et al., 2012). The recombinant GCSF manufactured in the bacterial system is non-glycosylated; GCSF produced in CHO cells is glycosylated and does not differ from G-CSF naturally made in humans. Both forms of recombinant hG-CSF are almost similar in pharmacological properties, although there are reports that the glycosylated form has a higher efficiency of mobilizing stem cells than Filgrastim and is more effective at lower doses (Ria et al., 2010; Sari et al., 2013). At that, the cost of GCSF production in CHO culture is significantly higher than in *E. coli* system. Therefore, efforts have been applied to develop alternative expression systems for recombinant GCSF production, including plant- based.

Recombinant GCSF was expressed in plastids of lettuce (Sharifi et al., 2013) and in nuclear-transformed tobacco plants (Sharifi et al., 2012). The level of GCSF accumulation in these studies is not reported. The hG-CSF was also successfully expressed in suspension - cultured tobacco and rice cells. In tobacco BY-2 cells, recombinant hG-CSF was expressed at 17.8 mg/l, the hG-CSF accumulation has been determined in the tissues of initial transgenic callus lines (Nair et al., 2014). Hong et al. (2006) produced hG-CSF in suspension culture of transgenic rice cells (*Oryza sativa* cv. Dong-jin). The expression of recombinant hG-CSF in callus lines amounted up to 185 μg g^−1^ of callus FW. The recombinant GCSF was able to secrete into the culture medium, its accumulation reached 2.5 mg l^−1^ of medium. Recombinant hG-CSF obtained in both studies retained specific biological activity, as shown in cell assays.

In our study, the accumulation of hG-CSF in the most productive lines WG-04 and WG-32 reaches 0.2% of TSP. Such a level of recombinant protein expression (a few of tenths of a percent) is common for nuclear-transformed plants (Lico et al., 2012; Shahid and Daniell, 2016). However, due to the high content of total protein in *Wolffia*, the yield of hG-CSF amounted up to 35 mg kg^−1^ of fresh weight, and this is a fairly good level. It should be mentioned that the cultivation conditions of transgenic *Wolffia* plants have not yet been fully optimized, so under the optimal conditions the hG-CSF yield could be significantly increased. In addition, it is of great interest to study the *Wolffia* recombinant proteins secretion into the culture medium on the example of GCSF; hence this will be the aim of our further research.

In our experiments we have expressed the human G-CSF in nuclear-transformed *Wolffia* plants without impact on plant morphology or growth rate. The accumulation of target hG-CSF protein in the best transgenic line reached 35 mg kg^−1^ FW. In conclusion the present study distinctly demonstrates the feasibility of recombinant proteins expression in nuclear-transformed *Wolffia* plants and opens the way for development of *Wolffia*-based expression systems.

## Author contributions

PK, AS, and MC: Obtaining transgenic lines of wolffia; AF, LS, and OK: Western blot analysis and ELISA of transgenic plants; AP Southern blot analysis; IT: Gene synthesis and plasmid construction; IC: Light microscopy; SD: Scientific adviser of this project.

### Conflict of interest statement

The authors declare that the research was conducted in the absence of any commercial or financial relationships that could be construed as a potential conflict of interest.

## Appendix

atggcgaagaggatcgcctccatgtcctcgctcctcctgattgcgctgctgtgcctcagctcccacctcgcgcaggccacgcctctgggccccgcctcctccctcccccagtccttcctcctgaagtgtctggagcaggtcaggaagatccaaggcgatggagccgccctccaggagaagctgtgcgcgacctacaagctgtgccaccccgaggaactcgtgctcctgggccattctctcggcattccgtgggcccccctgtcgtcttgtccttcgcaagccctgcagctcgccggatgcctctcccagctgcactcgggactcttcctctaccagggactcctccaagcgctcgagggcatctctcccgaactcggacctacgctcgacaccctccaactggacgtggcggatttcgccacgaccatctggcaacaaatggaagaactcggaatggcgcccgccctgcagcccactcaaggggcgatgcccgcgttcgcctccgcctttcagcggcgggccgggggagtgctggtggcgtcgcacctccagtcttttctcgaagttagctatcgcgtcctcaggcatctcgcccaaccg*tga*

Nucleotide sequence of hG-CSF for expression in *W. arrhiza* (the sequence of the signal peptide is highlighted in yellow, stop codon is italicized).
