# Supplementary material for: Development of Wolffia arrhiza as a Producer for Recombinant Human Granulocyte Colony-Stimulating Factor
Source: Front Chem. 2018 Jul 25;6:304. doi: 10.3389/fchem.2018.00304 (PMC6094986; doi:10.3389/fchem.2018.00304)
Supplement: Supplementary file 1 [file Table_1.DOCX]

**Contact Information**

Name:

Pavel Khvatkov

Contact Phone Number:

+79184076831

Contact Email:

Khvatkov1987@gmail.com

Affiliation:

Nikita Botanical Gardens – National Scientific Centre (NBG-NSC)

Russia, Republic of Crimea,Yalta, urban vil. Nikita, st. Nikita spusk 52, 298648

**Manuscript Information (if applicable)**

Title:

Development of Wolffia arrhiza as a producer for recombinant human granulocyte colony-stimulating factor

Journal:

Frontiers in Chemistry

Authors:

Pavel Khvatkov, Aleksey Firsov, Anastasiya Shvedova, Lyubov Shaloiko, Oleg Kozlov, Mariya Chernobrovkina, Alexander Pushin, Irina Tarasenko, Inna Chaban, Sergey Dolgov

**Species Identification Information**

Name Of Species:

*Wolffia arrhiza*

Morphological Classification (if applicable):

Water floating perennial; plurisonal, hemicosmopolite; hydrophyte. The root system is absent. Fronds are less than 1 mm in diameter of a juicy-green color. It reproduces vegetatively.

Molecular Classification:

atpF-atpH barcode:

psbK-psbI barcode:

AFLP-Lemna Genotype:

AFLP-Wolffia Genotype:

Other Sequence:

**Species Collection And Cultivation Information**

Date:

Cultivated *in vitro* since 03.12.2008.

Location:

(Provide information on site of collection. Include country, state/province, and city/town. Please be as specific as possible.)

For research, we obtained a population of Wolffia arrhiza (L.) Horkel ex Wimm from the collection of Main Botanic Garden of the Russian Academy of Sciences. The collection of the botanical garden plants collected from the western edge of the Kursk, shallow water of Mokwa technical pond.

Cultivation Information:

(Provide information on cultivation of clone since collection and how it is maintained. Mention if any genetic modifications or any other treatments have been performed on clone that may affect its natural physiology.)

The clone is cultivated under aseptic condition on SH agar medium. No genetic manipulation has been performed of this mother population.

**To which Duckweed collection are you able to submit your clone?**

(One of the goals of the RDSC is to have its registered clones available to the community to promote research and applications.)

RDSC

University Of Jena
